# Single Center Retrospective Analysis of Conventional and Radial TIG Catheters for Transradial Diagnostic Coronary Angiography

**DOI:** 10.1155/2015/862156

**Published:** 2015-09-08

**Authors:** Marc Vorpahl, Till Koehler, Jason Foerst, Spyridon Panagiotopoulos, Heinrich Schleiting, Klaus Koss, Gunda Ziegler, Hilmar Brinkmann, Melchior Seyfarth, Klaus Tiroch

**Affiliations:** ^1^Department of Cardiology, HELIOS Klinikum Wuppertal, Witten/Herdecke University, 42117 Wuppertal, Germany; ^2^Virginia Tech Carilion School of Medicine, Roanoke, VA 24016, USA

## Abstract

Current guidelines favor the radial approach for coronary angiography. Therefore, specialty radial diagnostic catheters were designed to engage both coronary arteries with a single device. However, it is unclear if single catheters are superior to conventional catheters. A retrospective analysis was performed of consecutive right radial coronary angiographies to determine catheter use, fluoroscopy time, radiation dosage, and consumption of contrast. Procedures were performed with a single TIG catheter or conventional catheters (CONV). Procedures with coronary artery bypass grafts or ventricular angiographies were excluded. 273 transradial procedures were performed successfully. 95 procedures were performed with CONV and 178 procedures with a TIG. Crossover to additional catheters was higher in TIG (15.2%) compared to CONV (5.3%, *p* = 0.02). Fluoroscopy time was comparable between CONV and TIG, without crossover (2.2 ± 1.2 min versus 2.3 ± 1.2 min; n.s.), however, greater in the case of crossover for CONV (5.8 ± 0.7) and TIG (7.6 ± 3.0; *p* = 0.0001). Radiation dosage was similar in CONV and the TIG, without crossover (1419 ± 1075, cGy*∗*cm^2^ versus 1690 ± 1138; n.s.), however, greater for CONV (2374 ± 620) and TIG (3733 ± 2281, *p* = 0.05) with crossover. Overall, the amount of contrast was greater in TIG (56 ± 13 mL) versus CONV (48 ± 3 mL; *p* = 0.0003). CONV femoral catheters may be the primary choice for radial approach.

## 1. Introduction

Nearly two decades after the original description [1], the transradial approach to coronary catheterization has become routine if not preferred throughout the global interventional community [2–7]. Recent international guidelines recommend the radial access for percutaneous coronary diagnostic and interventions even in acute coronary syndromes due to a significant reduction of major adverse events compared to femoral artery access [8–10]. Innovative catheter designs have been developed to allow diagnostic coronary angiography from the radial approach with a single catheter for both coronary arteries with the aim of reducing radial vasospasm, radiation dosage, and procedure time. Alternatively, conventional femoral approach catheters are also frequently used for the transradial access, for example, the Judkins left (JL) for the left coronary artery and the Judkins right (JR) or the Amplatz right I (AR I) catheter for the right coronary artery. The variation of the vascular anatomy and complexity of angles of the supra-aortic arteries remain a challenge for the radial approach in a significant percentage of patients.

The purpose of the study was to compare the usage and success of a single specialty catheter versus conventional (CONV) femoral catheters for diagnostic coronary angiography in a large single center's all comer population. We assessed the rate conversion from the intended primary catheters, fluoroscopy time, radiation dose, and consumption of contrast agent.

## 2. Materials and Methods

### 2.1. Patients

For this study, we evaluated patients undergoing transradial coronary angiography between June 2012 and June 2014. Patients with coronary artery bypass grafting (CABG), a left ventriculography, a left radial approach, need for conversion to femoral access, or percutaneous intervention were excluded from the current analysis. A total of 273 patients were eligible for inclusion.

### 2.2. Procedural Access Site Characteristics

Local standard procedure protocols demand a palpable right radial artery pulse and a nonpathologic Allen's test. Radial artery is punctured with a 21-gauge needle of the slightly elevated forearm and supine wrist. The standard right radial approach was performed with a Terumo Glidesheath Nitinol Kit. The radial artery was initially cannulated with a 21-gauge metal needle, and a 0.021 nitinol guide wire was inserted through the needle. An 11 cm 5F radial sheath with a sideport extension was then inserted with a shaped dilatator. To prevent arterial spasm, 1-2 mg of verapamil was administered through the sideport, and 2500 IU of heparin was given as an intra-arterial bolus. The sheath was removed immediately after the diagnostic procedure. Hemostasis was achieved by a radial compression device (TR Band TM, Terumo) with radial artery compression for 4 hours.

### 2.3. Diagnostic Catheters

**Figure 1:**
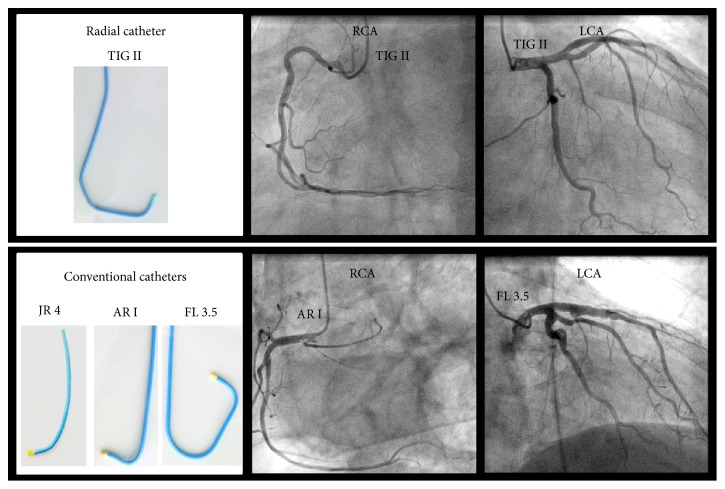
Design and performance of radial (TIG) and conventional (CONV) catheters.

The procedures were exclusively performed by experienced and board-certified angiographers with >500 radial diagnostic procedures. The usage of TIG or CONV catheters was at the discretion of the operator. The 5F Tiger II (3.5/4.0) diagnostic radial catheters (Terumo Corporation, Tokyo, Japan) are made of a polyurethane blend with an inner stainless steel wire net. Inner dimension is 0.047 inches. The distinct design of the catheter aims to provide the engagement of both coronary arteries with a single catheter (Figure 1).

Conventional catheters for the left coronary arteries are the 5F Judkins left (JL). The Judkins right (JR) and the Amplatz right I (ARI) (Cordis Corporation, Miami, FL) were used for the diagnostic of the right coronary artery (Figure 1). All diagnostic catheters were inserted through the 5F radial catheter sheath introducer.

### 2.4. Coronary Artery Cannulation and Diagnostic Procedure

A standard J-curve 0.035 guide wire (Radifocus M, Terumo, Japan) is used for the insertion and exchange of catheters. The diagnostic procedure had to include four standard views for the LCA and two for the RCA. In the case of failure to engage the coronary artery ostium, crossover to alternative CONV or TIG catheter is required to complete the diagnostic procedure.

### 2.5. Study End Points and Definitions

The primary end points of this study included an analysis of the time needed for the procedures for the CONV or the TIG group. The following data was assessed from the procedure: (1) inability to use intended catheters, (2) the total fluoroscopic time in minutes, (3) the radiation dosage in cGy*∗*cm^2^, and (4) the consumption of contrast medium in milliliter.

### 2.6. Data Collection and Statistical Analysis

Complete clinical, demographic, and angiographic characteristics were prospectively recorded on standardized forms by physicians or trained technicians and entered into our study database.

Categorical variables were compared by chi-square test or Fisher exact test as appropriate. Continuous variables were analyzed by an independent-samples *t*-test. Results are reported as the mean ± SD. Statistical analysis was performed with the SPSS, version 11.5, software package (SPSS, Chicago, IL). A *p* value of <0.05 was considered statistically significant.

## 3. Results

### 3.1. Study Population and Diagnostic Catheter Characteristics

We analyzed a total of 237 consecutive diagnostic coronary angiography cases from the right radial approach. Of these, 178 cases were performed with a single catheter (TIG) and 95 cases were performed with conventional catheters (CONV) (Figure 1). The patients of the TIG group and the CONV groups were overall well balanced for gender, age, risk factors, ejection fraction, height, weight, body surface area, and the body mass index. The overall BMI (kg/m^2^) ranged from 28 to 36 in our study population which is considerably higher than the defined normal BMI range. However, we observed a significant difference for the extent of vessels diseased. Diagnostic angiograms were successfully achieved in all cases via the radial approach (Table 1).

**Figure 2:**
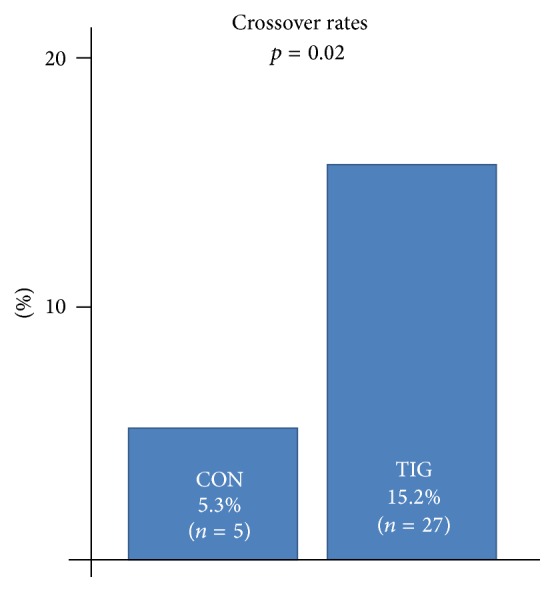
Crossover rates in conventional (CON) catheters versus radial (TIG) catheters.

Supplemental Judkins or Amplatz catheters were used to complete the diagnostic procedure in case of failure to engage the diagnostic catheters appropriately into the left or right coronary artery. Utilization of supplemental catheters (crossover) was significantly greater in the TIG group (15.2%, *n* = 27) versus the CONV group (4.3%, *n* = 5, *p* = 0.02) (Table 1 and Figure 2). There were no conversions to a femoral approach with all cases achieving adequate diagnostic angiograms via the radial approach. There were no angiographic or clinical complications.

### 3.2. Procedural Characteristics

Fluoroscopy time was significantly greater in TIG (2.4 ± 1.5 min) versus CONV (3.1 ± 2.5 min; *p* = 0.01). This was mainly related to the greater utilization of supplemental catheters (crossover) in TIG. Fluoroscopy times after crossover were significantly greater in CONV (5.8 ± 0.7, *p* = 0.0001) and TIG (7.6 ± 3.0 min, *p* = 0.0001). Fluoroscopy time was comparable between CONV and TIG, without crossover (2.2 ± 1.2 min versus 2.3 ± 1.2 min; n.s.) (Table 2).

**Figure 3:**
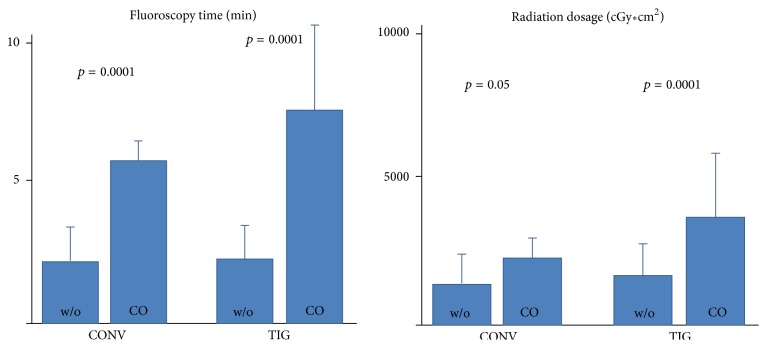
Fluoroscopy time and radiation dosage in conventional catheters versus radial catheters.

We observed the same finding for the radiation dosage. Radiation dosage was significantly greater in TIG (2000 ± 1550, cGy*∗*cm²) versus CONV (1468 ± 1075, cGy*∗*cm²; *p* = 0.0003). This was again mainly related to the greater utilization of supplemental catheters (crossover) in TIG. Radiation dosages after crossover were significantly higher in both TIG and CONV groups after crossover (both *p* < 0.001), both also significantly higher after crossover in the TIG group (3733 ± 2281, cGy*∗*cm²) compared to the CONV group (2372 ± 620 cGy*∗*cm²). Radiation dosage was comparable between CONV and TIG, without crossover (1419 ± 1075 versus 1690 ± 1138; n.s.) (Table 2 and Figure 3).

Consumption of contrast medium was greater in TIG (56 ± 19 mL) versus CON (48 ± 13 mL; *p* = 0.0003). This finding was also observed for TIG w/o crossover (54 ± 17 mL) versus CONV (47 ± 13 mL; *p* = 0.0009). Consumption was greatest in the crossover groups for CONV (57 ± 13 mL) and TIG (69 ± 22 mL).

## 4. Discussion

Transradial approach is a safe and feasible alternative to transfemoral access in coronary angiography. As of today, there is no standard for the optimal choice of radial catheters in daily clinical practice. However, the actual EAPCI/ESC consensus publication on the radial approach in percutaneous cardiovascular interventions favors the combination of conventional catheters like Judkins left for LCA and Judkins right or Amplatz right for RCA [8]. Special multipurpose catheters for transradial access like Tiger II are suggested as further options. There are very few studies comparing catheter shape and procedural success rate for transradial approach to coronary angiography. In 2006 Kim et al. [11] made a comparison of the Tiger II and Judkins left catheter by measuring procedure time and fluoroscopy time. They demonstrated superior right coronary angiographic quality with the Tiger II and a significant benefit in procedure and fluoroscopy time but no difference for left coronary angiographic quality. Overall, the fluoroscopy time in the prospective randomized trial of Kim et al. was significantly lower in TIG (1.55 min) versus CONV (2.3 min) [11]. This was lower than our findings in an all-comer real world setting with 2.4 min for CONV and 3.1 min for the TIG. Also of note, our study patients had a BMI (kg/m^2^) between 28 and 36 which is considerably higher than the considered normal BMI range. This is most likely related to a local selection bias to perform the radial approach particularly in obese patients to avoid the femoral access adverse bleeding events.

We observed a crossover rate of 15%, which is higher compared to the 6.5% reported by Tebet et al. [12], but lower than the 44% crossover rate reported by Langer et al. [13]. In the study by Tebet et al., the success rate and procedural duration were similar for the overall population, with an advantage for the TIG catheter. However, the crossover rate was also higher for TIG catheter compared to conventional catheters. In patients with multivessel disease, crossover was more frequent in the TIG group. This may be related to selection bias. However, another possible explanation might be the need to achieve improved image quality for the correct assessment of complex anatomy, requiring additional catheter changes.

This and other publications highlight the importance of catheter choice and the individual training of the operator as a key role for a successful procedure dealing with varying individual anatomical proportions. Our data suggests that when there is no need for conversion there is no significant difference between the specialty transradial TIG catheter and the conventional catheter approach with JL and AR I in regard to fluoroscopy time, radiation dosage, and contrast volume. However, the need for converting from the TIG II or JL/AR1 to other conventional catheters (crossover) resulted in a significant increase in all parameters measured. This finding was remarkably higher in the TIG group suggesting that CONV catheters may be the best primary choice for the radial approach.

## 5. Limitations

We were not able to assess rates of vasospasm and radial artery occlusion after the radial diagnostic procedure due to the retrospective study design. However, a retrospective analysis may be susceptible for bias in data selection and analysis. The findings from our study are hypothesis generating and may need further validation by a larger prospective randomized trial. The analysis of consecutive patients does represent the “real world” in our large interventional centre.

The TIG catheter was the only analysed multifunctional catheter in this study. The result of our study does not apply to other multifunctional radial catheters (e.g., Kimny catheter) or to other guiding catheters.

The studied conventional catheters were manufactured by Cordis, while the multifunctional TIG catheter was obtained from Terumo. The catheters might therefore differ in regard to specific design and material.

## 6. Conclusion

Conventional catheters may be the primary choice for diagnostic coronary angiography from the right radial approach.

## Figures and Tables

**Table 1:** Baseline characteristics.

	CONV total	TIG total	*p* value	CONV w/o CO	TIG w/o CO	*p* value	CONV CO	TIG CO	*p* value
	(*n* = 95)	(*n* = 178)		(*n* = 90)	(*n* = 151)		(*n* = 5)	(*n* = 27)	
Female (*n*/%)	41/43.2	73/42.1	n.s.	39/43.3	65/43.0	n.s.	2/40.0	8/29.6	n.s.
Age (y)	71.1 ± 12.3	68.8 ± 12.0	n.s.	71.1 ± 12.3	69.2 ± 12.4	n.s.	71.4 ± 12.8	66.5 ± 9.5	n.s.
Diabetes (*n*/%)	29/30.5	46/25.8	n.s.	27/30.0	38/25.2	n.s.	2/40.0	8/29.6	n.s.
Hypertension (*n*/%)	81/85.3	146/82.0	n.s.	77/85.6	121/80.1	n.s.	4/80.0	25/92.6	n.s.
Hyperlipidemia (*n*/%)	49/51.6	83/46.6	n.s.	48/53.3	69/45.7	n.s.	2/40.0	14/51.9	n.s.
Smoker (*n*/%)	29/30.5	53/29.8	n.s.	28/31.1	43/28.5	n.s.	1/20.0	10/37.0	n.s.
Vessel disease (0/1/2/3)	36/25/15/23	96/37/17/35	0.02	33/24/14/23	81/31/13/31	0.02	3/1/1/0	15/6/4/4	n.s.
Ejection fraction (%)	55.2 ± 12.2	57.6 ± 10.8	n.s.	55.2 ± 12.2	57.6 ± 10.9	n.s.	54.6 ± 13.2	57.6 ± 11.1	n.s.
Height (m)	1.70 ± 0.09	1.71 ± 0.10	n.s.	1.70 ± 0.09	1.71 ± 0.10	n.s.	1.63 ± 0.07	1.72 ± 0.10	n.s.
Weight (kg)	82.7 ± 17.7	85.7 ± 20.2	n.s.	82.2 ± 17.9	84.7 ± 20.5	n.s.	93.4 ± 9.7	91.6 ± 17.4	n.s.
BSA m^2^	1.94 ± 0.21	1.98 ± 0.25	n.s.	1.94 ± 0.21	1.97 ± 0.24	n.s.	2.01 ± 0.09	2.02 ± 0.28	n.s.
BMI	28.8 ± 6.3	29.4 ± 6.9	n.s.	28.4 ± 6.0	29.1 ± 6.9	n.s.	36.4 ± 7.3	31.2 ± 6.8	n.s.

CONV: conventional catheter group; TIG: TIG catheter group; w/o: without; CO: crossover.

**Table 2:** Fluoroscopy time, dosage, and contrast medium for CONV versus TIG catheters.

	CONV total	TIG total	*p* value	CONV w/o CO	TIG w/o CO	*p* value	CONV CO	TIG CO	*p* value
	(*n* = 95)	(*n* = 178)		(*n* = 90)	(*n* = 151)		(*n* = 5)	(*n* = 27)	
Fluoroscopy time (min)	2.4 ± 1.5	3.1 ± 2.5	0.01	2.2 ± 1.2	2.3 ± 1.2	n.s.	5.8 ± 0.7	7.6 ± 3.0	n.s.
Radiation dosage (cGy*∗*cm²)	1468 ± 1075	2000 ± 1550	0.003	1419 ± 1075	1690 ± 1138	n.s.	2372 ± 620	3733 ± 2281	n.s.
Contrast agent (mL)	48 ± 13	56 ± 19	0.0003	47 ± 13	54 ± 17	0.0009	57 ± 13	69 ± 22	n.s.

CONV: conventional catheter group; TIG: TIG catheter group; w/o: without; CO: crossover.

## Abbreviations

AR I: Amplatz right I catheter
BMI: Body mass index
BSA: Body surface area
CABG: Coronary artery bypass graft
cGy*∗*cm^2^: Centi-Gray*∗*centimeter^2^ dose-area product
CO: Crossover
CONV: Conventional catheter group
F: French
JL: Judkins left catheter
JR: Judkins right catheter
LCA: Left coronary artery
mL: Milliliter
min: Minute
RCA: Right coronary artery
sec: Seconds
TIG II: Angiographic Optitorque Tiger diagnostic catheter
TIG: TIG catheter group
w/o: Without.
